# Transient signal generation in a self-assembled nanosystem fueled by ATP

**DOI:** 10.1038/ncomms8790

**Published:** 2015-07-21

**Authors:** Cristian Pezzato, Leonard J. Prins

**Affiliations:** 1 Department of Chemical Sciences, University of Padova, Via Marzolo 1, 35131 Padova, Italy

## Abstract

A fundamental difference exists in the way signal generation is dealt with in natural and synthetic systems. While nature uses the transient activation of signalling pathways to regulate all cellular functions, chemists rely on sensory devices that convert the presence of an analyte into a steady output signal. The development of chemical systems that bear a closer analogy to living ones (that is, require energy for functioning, are transient in nature and operate out-of-equilibrium) requires a paradigm shift in the design of such systems. Here we report a straightforward strategy that enables transient signal generation in a self-assembled system and show that it can be used to mimic key features of natural signalling pathways, which are control over the output signal intensity and decay rate, the concentration-dependent activation of different signalling pathways and the transient downregulation of catalytic activity. Overall, the reported methodology provides temporal control over supramolecular processes.

## Abstract (web summary)

Natural and synthetic systems have fundamentally different approaches to signal generation. Here, the authors report a strategy that enables transient signal generation in a self-assembled system and show that it can be used to mimic several key features of natural signalling pathways.

## Graphical abstract

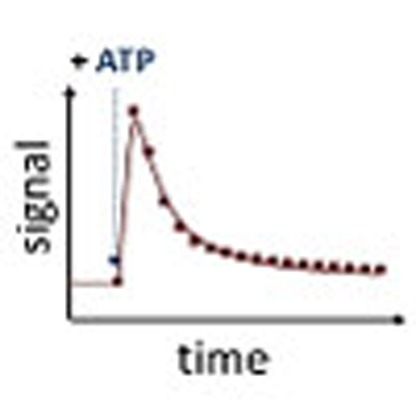

Communication is an essential feature of all living systems as it permits coordination, organization and adaptation^1^. To achieve this, nature has evolved elaborate signalling pathways relying on circular enzymatic networks to regulate all intra and extracellular functions^2^,^3^. In such networks, a trigger can up or downregulate an enzymatic cascade reaction leading to the transient generation of an output signal, after which the system returns to the original state. The circular nature of enzymatic networks implies that signal generation is a process that requires energy consumption. Over the past decades, chemists have developed a wealth of supramolecular sensory systems able to generate an output signal in response to an external trigger^4^,^5^,^6^,^7^,^8^. A common feature of these systems is that signal generation is thermodynamically driven, that is, the system adapts to a trigger-induced change in the energetic landscape developing into a new, energetically more favourable, resting state. This change is accompanied with a change in a property (fluorescence, current, catalysis, solubility and so on) that can be measured and correlated to the intensity of the trigger. This approach is exemplified in Fig. 1a (step i) for an indicator-displacement assay in which an analyte displaces a receptor-bound indicator^9^. The energy cost required for dissociation of the indicator (Δ*G*°_diss_) is more than compensated for by the energy gain resulting from formation of the receptor–analyte complex (Δ*G*°_ass_) (Fig. 1b). Overall this process is energetically downhill and generates a signal that is stable in time. For applications in sensing, this is obviously a highly attractive property and, consequently, a wide variety of supramolecular sensing systems have been developed based on this principle^4^,^5^,^6^,^7^,^8^,^10^. However, the absence of a return to the original state, which would require an amount of energy equal to Δ*G*°_ass_−Δ*G*°_diss_, makes it fundamentally different from the way nature deals with signal generation. Indeed, there is currently an enormous interest in the development of synthetic systems that require energy consumption to remain in a functional state^11^. Such systems have properties that are more similar to living systems and are expected to offer new applications in the field of materials and nanotechnology^12^. Examples of dissipative self-assembled systems^13^,^14^,^15^, transient catalysts^16^, molecular transport systems^17^ and molecular motors^18^,^19^,^20^ are emerging.

Here we demonstrate a general strategy that permits transient signal generation in a self-assembled system. The approach relies on the addition of an additional step to the scheme depicted in Fig. 1a (step ii) that provides the necessary energy (Δ*G*°_ass_−Δ*G*°_diss_) to convert the system back to the original state. It is shown that this gives the possibility to perform multiple signalling cycles with the same system and, importantly, allows for temporal control over signal intensity. It is then demonstrated that this approach can be used to mimic features of natural signalling pathways, such as the activation of one or two signalling pathways depending on the initial trigger concentration and the transient regulation of catalytic activity. A related strategy has been previously applied in the context of enzyme assay development^21^ and for studying the conformational dynamics of a protein^22^. Also an entirely different approach towards (irreversible) transient signal generation has been reported^23^.

## Results

### Transient signal generation fuelled by ATP

**Figure 1:**
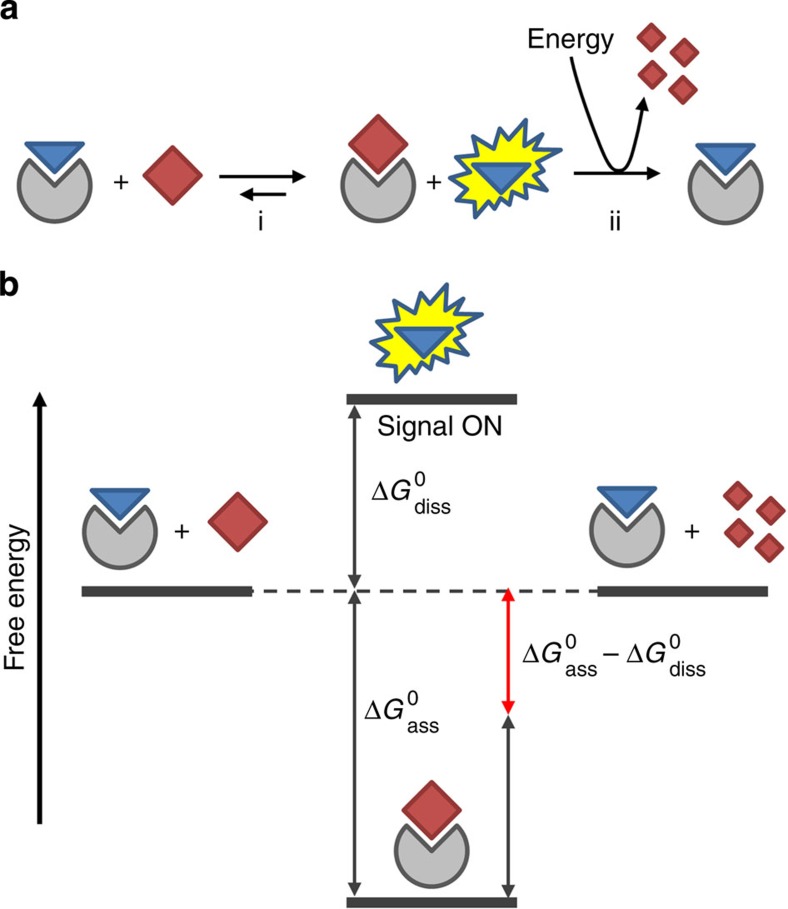
Transient signal generation. (**a**) Schematic representation of an indicator-displacement assay (step i). An additional energy-consuming step (ii) is required to revert the system back to the original state. (**b**) Schematic energy diagram indicating the changes in free energy related to steps i and ii of the indicator-displacement assay depicted in **a**.

**Figure 2:**
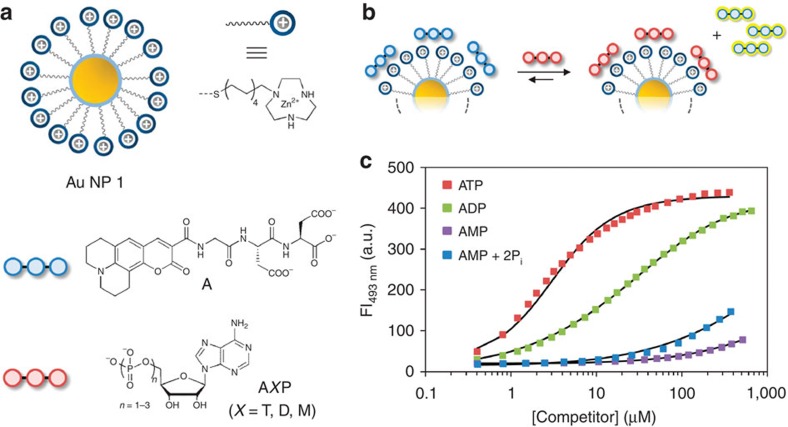
Displacement studies. (**a**) Schematic representations of the molecules. (**b**) Displacement of probe **A** from Au NP **1** on the addition of a competitor. (**c**) Fluorescent intensity at 493 nm as a function of the concentration of competitor. The solid lines represent the best fit to model. Experimental conditions: [TACN·Zn^2+^]=10±1 μM, [**A**]=3.7 μM, [HEPES]=10 mM, pH 7.0, [CaCl_2_]=1.0 mM, 37 °C, *λ*_ex, **A**_=450 nm, *λ*_em, **A**_=493 nm, slits=2.5/5.0 nm.

**Figure 3:**
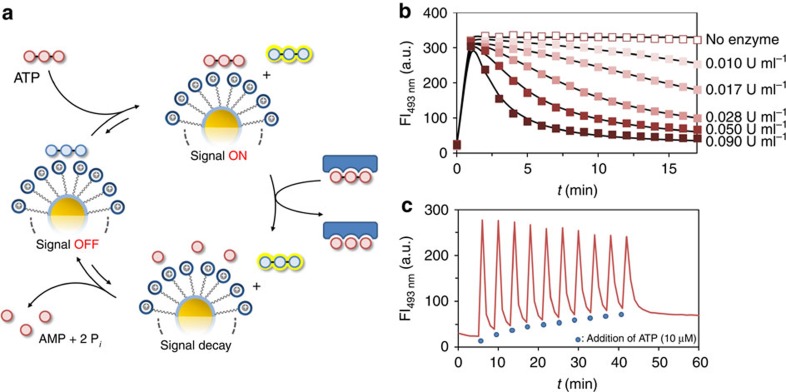
Transient signal generation fuelled by ATP. (**a**) Schematic representation of the system for transient signal generation. (**b**) Fluorescent intensity at 493 nm as a function of time on the addition of ATP (10 μM) to Au NP **1** ([TACN·Zn^2+^]=10±1 μM) and **A** (3.7 μM) in the presence of different concentrations of potato apyrase. The solid lines represent fits to the kinetic model shown in Fig. 4. (**c**) Fluorescent intensity at 493 nm on 10 repetitive additions of ATP (10 μM) to a solution of Au NP **1** ([TACN·Zn^2+^]=10±1 μM) and **A** (3.7 μM) in the presence of potato apyrase (0.3 U ml^−1^).

Our system relies on the strong interaction between oligoanions and the cationic surface of Au NP **1**, which are gold nanoparticles (*d*=1.8±0.4 nm) covered with hydrophobic C9-thiols terminating with a 1,4,7-triazacyclononane (TACN)·Zn^2+^ head group (Fig. 2a)^24^. Previous studies have shown that binding occurs under saturation conditions even at low micromolar concentrations in aqueous buffer (see the Methods section and Supplementary Fig. 1 for more details)^25^,^26^. These studies also showed that the strength of interaction strongly depends on the number of negative charges present in the oligoanions. This emerges clearly from a series of displacement experiments in which increasing amounts of A*X*P (*X*=T, D, M) are added to Au NP **1** ([TACN·Zn^2+^]=10±1 μM) saturated with fluorescent probe **A** (3.7 μM, *λ*_ex_=450 nm, *λ*_em_=493 nm) (Fig. 2b). When bound to Au NP **1**, the fluorescence of **A** is quenched by the gold nanoparticle, but is turned on upon its displacement by nucleotides A*X*P (*X*=T, D, M). This attractive property of gold nanoparticles has been at the basis of numerous indicator-displacement assays for a variety of (bio)analytes^27^,^28^,^29^,^30^. Measurement of the fluorescence intensity (FI) as a function of the concentration of A*X*P provides information on the relative affinity, *K*_rel_, of A*X*P for Au NP **1** compared with **A** (Fig. 2c). Fitting of the measured curves yielded *K*_rel_ values of 1.8, 9.2 × 10^−2^ and 2.8 × 10^−4^ for ATP, ADP and AMP, respectively, demonstrating the importance of multivalent interactions. In the past, we have exploited this for the development of assays based on an analyte-induced change in the thermodynamic equilibrium (analogous to step i in Fig. 1)^31^,^32^. However, we were intrigued by the question of whether this system would be able to generate a fluorescent signal that is transient in nature. This would rely on the initial displacement of **A** by the strong competitor ATP after which the irreversible destruction of ATP into weaker competitors would result in re-formation of the complex between **A** and Au NP **1**, and thus a disappearance of the signal (Fig. 3a). The enzyme potato apyrase very efficiently hydrolyses ATP into AMP and 2 equiv. of inorganic phosphate P_i_, which makes it very suitable for the purpose given above^33^. We first verified that the presence of the enzyme had no effect on the stability of the complex between **A** and Au NP **1** (see Supplementary Fig. 2) and determined the *K*_rel_-value of the AMP+2P_i_ mixture rather than just AMP (Fig. 2c). As expected, a slightly higher value (1.6 × 10^−3^) was obtained, but still around 3 orders of magnitude lower than that of ATP (see the Methods section and Supplementary Figs 3 and 4 for full details about the displacement studies).

Kinetic measurements showed that in the absence of enzyme, the addition of ATP (10 μM) to a solution of **A** and Au NP **1** led to a rapid increase in FI, which then remained constant in time (Fig. 3b). We were very pleased to observe that repetition of the same experiment in the presence of increasing amounts of enzyme (from 0.01 up to 0.09 U ml^−1^) resulted in the same initial increase in FI, but this time followed by a signal decay with rates depending on the concentration of enzyme (Fig. 3b). At the highest concentration of enzyme, the FI returned to the starting value after just 15 min. The reproducibility of transient signal generation by the system was demonstrated by performing 10 cycles with the same sample adding new batches of ATP (10 μM) each time the signal had returned to the starting value (Fig. 3c). It is noted that the higher fluorescent intensity after 10 cycles (69 a.u.) originates from the accumulation of waste (AMP+2P_i_) in the system (72 a.u. expected for 100 μM of AMP and 200 μM of P_i_ based on the displacement curves).

### Signal–response curve and kinetic model

**Figure 4:**
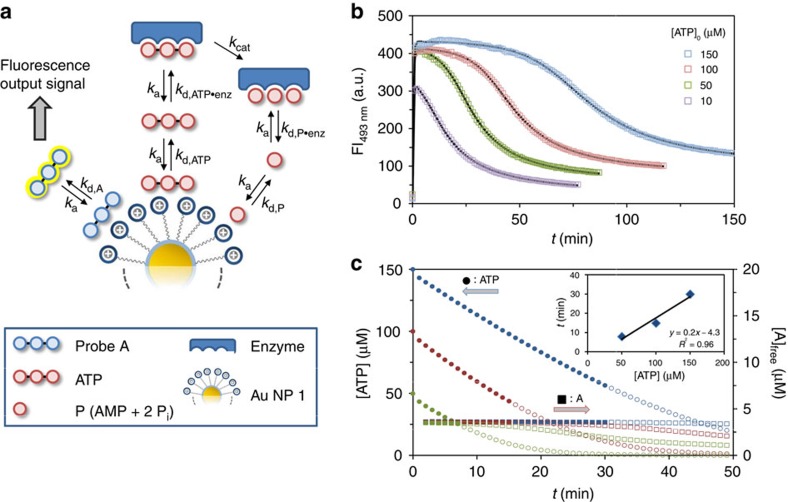
Signal–response curve and kinetic model. (**a**) Kinetic model used for fitting the experimental data. (**b**) Fluorescent intensity at 493 nm as a function of time on the addition of different amounts of ATP to a solution containing Au NP **1** ([TACN·Zn^2+^]=10±1 μM), probe **A** (3.7 μM) and a constant concentration of potato apyrase (0.017 U ml^−1^). The solid lines represent the fit to a kinetic model. (**c**) Plots of the concentration of ATP (left axis) and free **A** (right axis) as a function of time for three different initial concentrations of ATP. The solid points indicate the period at which the concentration of free **A** is constant (<2% decrease with respect to the maximum value). The inset gives the duration of the time interval with a steady concentration of free **A** as a function of the initial amount of ATP.

The most important feature of any signalling system is the signal–response curve, as it correlates the output signal to the intensity of the input signal. We determined the response curves of our system at four different concentrations of ATP (10, 50, 100 and 150 μM) at a constant concentration of the enzyme (0.017 U ml^−1^). Measurement of the FI as a function of time clearly showed strong differences between the four samples (Fig. 4b). In all cases, the addition of ATP caused a rapid increase of the FI. The maximum intensity was lower for [ATP]_0_=10 μM (FI_493_=306 a.u.), compared with the other three samples which reached a nearly identical intensity of ∼410 a.u. These values correspond to the fluorescence intensities measured in the competition experiments between probe **A** and ATP (Fig. 2c). The nature of the displacement curve indeed confirms that a FI of ∼410 a.u. is the maximum intensity of the output signal that can be generated by the system as it represents the situation at which (nearly) all of probe **A** is displaced from Au NP **1**. However, although the addition of 50, 100 or 150 μM of ATP gives the same initial signal, the signal decay is much different, being much slower for high concentrations of ATP. Although this can be intuitively explained based on the displacement curve, we were interested in a quantitative description of the signal generation process to have detailed information on the concentration of all species in the system. For that purpose, we developed a model taking into account all relevant kinetic processes that occur within the system (Fig. 4a, see Methods section and Supplementary Fig. 5 for more details). The relative affinities, *K*_rel_, were used to define the dissociation rate constants of all complexes involving Au NP **1**, assuming that all association rate constants are very high. Dissociation rate constants for the complexes involving the enzyme were varied to fit the experimental data to the model. As illustrated by the solid lines the model neatly describes the experimental data (Fig. 4b). Analysis of the concentrations of ATP and **A** as a function of time illustrates the most interesting feature of this system, which is the possibility to develop a steady fluorescent signal with a duration that depends on the initial concentration of ATP. Interestingly, during the period in which the concentration of free **A**, and thus the output signal, remains constant (<2% decrease), a significant consumption of ATP takes place (see filled symbols in Fig. 4c). However, not until the concentration of ATP falls below a certain threshold value at which **A** can effectively compete with the remaining ATP for binding to Au NP **1**, the signal starts to decay. The correlation between the duration of this lag time and the initial concentration of ATP demonstrates that it is possible to control this time interval, which may last up to 30 min (inset Fig. 4c). As will be discussed in the next section, signalling pathways in nature rely heavily on this kind of control mechanism.

### ATP-dependent activation of two different signals

**Figure 5:**
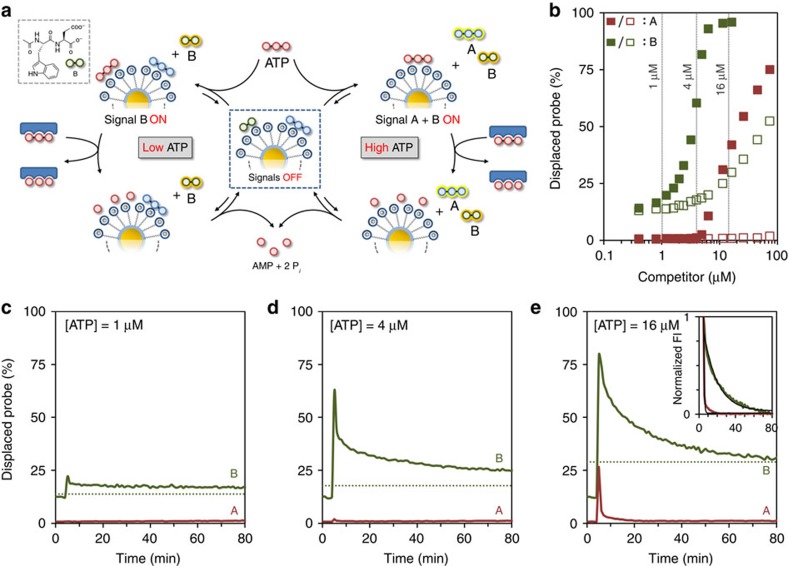
ATP-dependent activation of two different signals. (**a**) Schematic representation of the ATP-dependent transient generation of one or two signals. (**b**) Amount of displaced probe **A** (red) or **B** (green) as a function of the concentration of either ATP (filled squares) or the waste mixture AMP+2P_i_ (empty squares). (**c**–**e**) Amount of displaced probe **A** (red) and **B** (green) as a function of time on the addition of ATP at 1 (**c**), 4 (**d**) or 16 μM (**e**) concentrations. The dotted lines mark the expected end value expected for probe **B** in the presence of the respective amount of waste formed. The inset in **e** gives the normalized change in FI as a function of time for the two probes and demonstrates the much faster decay of the signal originating from the high affinity prove **A**. In all these experiments probe **A** was excited at 370 nm (rather than at 450 nm) to obtain similar fluorescence intensities for probes **A** and **B**, thus permitting a monitoring of both probes with the same slit widths. Experimental conditions: [TACN·Zn^2+^]=20±1 μM, [**A**]=[**B**]=0.5 μM, [HEPES]=10 mM, pH 7.0, [CaCl_2_]=1.0 mM, [potato apyrase]=0.3 U ml^−1^, 37 °C. *λ*_ex, **A**_=370 nm, *λ*_em, **A**_=493 nm, *λ*_ex, **B**_=280 nm, *λ*_em, **B**_=360 nm, slits=10/10 nm.

Having developed a robust system for transient signal generation, we were curious to find out whether it would be possible to mimic other features of natural signalling pathways. In particular, we were inspired by the purinergic signalling pathway as it bears a strong resemblance to our system. The purinergic signalling pathway is involved in the regulation of various physiological functions in the circulatory^34^, immune^35^ and nervous system^36^, among others. It operates through the activation of purinergic receptors in the cell membrane by extracellular nucleotides and nucleosides. It is known that all receptors belonging to the P2X family, which is one out of the three known distinct classes of purinergic receptors, respond only to ATP^37^. However, they require different concentrations of ATP for activation and exhibit different desensitization kinetics. For example, the P2X1 receptor has a high affinity for ATP (*K*_D_≈0.01–1 μM) and a desensitization rate <1 s, whereas activation of the P2X7 receptor requires ATP concentrations >100 μM but has a much slower desensitization rate (>20 s)^37^. Thus, the overall response of the signalling pathway (both in terms of output signal diversity and decay rate) is dependent on the intensity of the trigger (ATP). We argued that this property could be mimicked by adding a second fluorescent probe **B** (dipeptide Ac-WD-OH) to the system (Fig. 5a). Dipeptide **B** has a much lower affinity and surface saturation concentration (SSC) compared with **A** (0.6±0.1 μM for [TACN·Zn^2+^]=10±1 μM, see Supplementary Fig. 6) and, in addition, has a fluorescent tryptophan moiety (*λ*_ex_=280 nm, *λ*_em_=360 nm), which can be monitored independently from probe **A**. The displacement of probes **A** (0.5 μM) and **B** (0.5 μM) from Au NP **1** by either ATP or the waste mixture AMP+2P_i_ was studied at a slightly higher concentration of Au NP **1** ([TACN·Zn^2+^]=20±1 μM) to ensure that both probes were initially (nearly) quantitatively bound. As a result of the lower valency of probe **B** the margin between ATP and the waste mixture is much reduced. Nonetheless, the difference between probes **A** and **B** is sufficiently large to test the abovementioned hypothesis (Fig. 5b). In particular, three concentrations of ATP were identified at which the system was expected to generate a different response: 1 μM of ATP should not result in any signal, 4 μM of ATP should generate a signal from **B** but not **A**, whereas at 16 μM of ATP signals from both **A** and **B** should emerge. It is noted that higher concentrations of ATP could not be used because the accumulation of waste would block the re-formation of the complex between **B** and Au NP **1** after ATP hydrolysis. Also, it was found that high concentrations of ATP (>20 μM) caused a decrease in the fluorescence intensity of free **B**. Transient signal generation by the system was studied by measuring the fluorescence intensities of probe **A** and **B** in time on the addition of ATP at 1, 4 or 16 μM concentrations to the system in the presence of potato apyrase (Fig. 5c–e). The obtained curves clearly demonstrate that, just as in the purinergic signalling pathway, the initial concentration of ATP determines whether none, one or two signals are generated. It is noted that the amount of displaced **A** on the addition of 16 μM (27%) is lower than that expected based on the displacement assays (42%, Fig. 5b). This is caused by the hydrolysis of ATP by the enzyme in the time between the addition of ATP and the continuation of the measurements. Importantly, the system also displays a correlation between probe affinity and signal duration (see inset of Fig. 5e). Thus, activation of probe **A** requires high concentrations of ATP, but the resulting signal decays rapidly (*k*_obs_=1.5 min^−1^). On the other hand, low concentrations of ATP are sufficient to activate probe **B**, but that signal persists for a much longer time (23 × , *k*_obs_=0.07 min^−1^). The inverted relation between binding affinity and signal duration originates directly from the competition between the probes and ATP for binding to Au NP **1**. For example, low-affinity probe **B** requires a near-complete hydrolysis of ATP to be able to return to Au NP **1**. Here this takes a particular long time because the ATP hydrolysis by the enzyme slows down significantly on depletion of ATP.

### Transient downregulation of the catalytic activity of Au NP **1**

**Figure 6:**
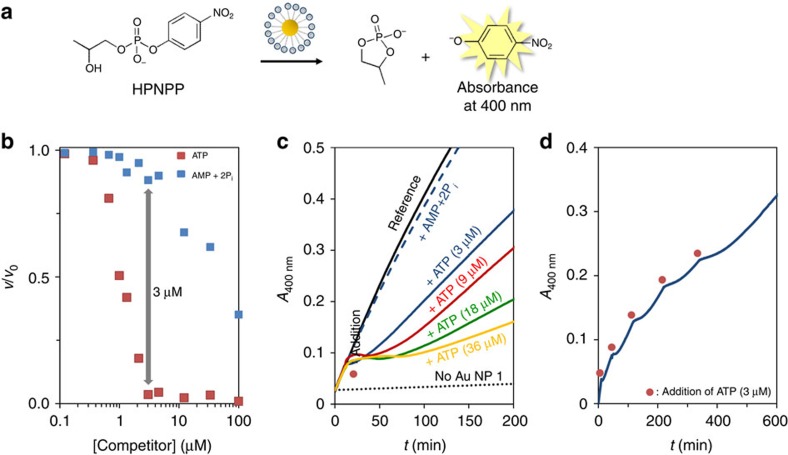
Transient downregulation of the catalytic activity of Au NP 1. (**a**) Catalysis of the transphosphorylation of HPNPP by Au NP **1**. (**b**) Relative reaction rates (*ν*/*ν*_0_) as a function of the concentration of either ATP or the mixture AMP+2P_i_. Experimental conditions: [TACN·Zn^2+^]=10±1 μM, [HPNPP]=1 mM, [HEPES]=10 mM, pH 7.0, [CaCl_2_]=1.0 mM, 37 °C. (**c**) Absorbance at 400 nm (originating from the reaction product *p*-nitrophenolate) as a function of time for different mixtures. The red dot indicates the time at which inhibitors were added. The lower slope after reactivation of Au NP **1** at higher concentrations of ATP is caused by the higher concentrations of produced waste (see the inhibition by different concentrations of the waste mixture given in Supplementary Fig. 8). Experimental conditions: [TACN·Zn^2+^]=10±1 μM, [HPNPP]=1 mM, [HEPES]=10 mM, pH 7.0, [CaCl_2_]=1.0 mM, [potato apyrase]=0.06 U ml^−1^, 37 °C. (**d**) Absorbance at 400 nm as a function of time on the sequential additions of ATP (5 × 3 μM). The red dots indicate the time of addition. Experimental conditions: same as for **c**.

Another feature of natural signalling pathways is that they consist of cascades of enzymatic reactions that are either up or downregulated by an initial trigger. This permits the system to transform a weak input signal into a strong output signal by means of catalytic signal amplification. We were interested to find out whether we could exploit the potato apyrase-driven consumption of ATP for the transient regulation of the catalytic activity of Au NP **1**. Previous studies have shown that Au NP **1** and analogues are highly efficient catalysts for the transphosphorylation of 2-hydroxypropyl-4-nitrophenylphosphate (HPNPP), which is an RNA-model compound (Fig. 6a)^38^,^39^. These kinds of catalytic nanosystems have been referred to as nanozymes, for the fact that the reaction rate follows Michaelis–Menten reaction kinetics^38^. This implies that catalysis is described by a binding event (defined by the dissociation constant *K*_M_) followed by a chemical reaction (defined by the first-order rate constant *k*_cat_). For the transphosphorylation of HPNPP by Au NP **1**, values for *K*_M_ and *k*_cat_ of 0.25 mM and 1.4 × 10^−3^ s^−1^, respectively, were determined (see the Methods section and Supplementary Fig. 7). As for enzymes, the catalytic activity of Au NP **1** is inhibited in the presence of species able to compete with HPNPP for binding^25^. Indeed, kinetic studies at varying concentrations showed that ATP is a very effective inhibitor already at low micromolar concentrations (Fig. 6b). However, a significantly lower inhibitory effect of the AMP+2P_i_ waste mixture was observed (Fig. 6b). This difference raised the possibility to temporarily downregulate the catalytic activity of Au NP **1** by ATP in the presence of potato apyrase. This was verified by adding 3 μM of ATP to a solution containing Au NP **1** (10 μM), HPNPP (1 mM) and potato apyrase (0.06 U ml^−1^). This caused an immediate inhibition of the transphosphorylation reaction evidenced by the constant absorbance at 400 nm (Fig. 6c). However, after a period of ∼5 min, the absorbance started to increase again indicating the reactivation of Au NP **1**. It is noted that even after prolonged times the catalytic activity of Au NP **1** was not restored to the level expected in the presence of AMP+2P_i_ (see dotted line in Fig. 6c). This could indicate that potato apyrase is inhibited by HPNPP causing a further reduction of the already slow hydrolysis rate of ATP at these concentrations. Nonetheless, the observation that the duration of the inhibition lag time depends on the amount of ATP added demonstrated that ATP indeed functions as transient regulatory element for the downregulation of Au NP **1** (Fig. 6c). The transient nature emerged also in a clear manner from a kinetic experiment in which five cycles were performed by sequentially adding ATP (3 μM each) to the mixture of Au NP **1,** HPNPP and the enzyme (Fig. 6d).

## Discussion

In conclusion, we have developed a strategy that permits transient signal generation in a self-assembled system. Signal formation is triggered by an input in the form of ATP, the consumption of which returns the system back to the original state. The initial concentration of ATP regulates the intensity of the output signal and its duration and determines also whether one or two signals are generated in the case where two reporter molecules are used. The same mechanism is evoked when applied to the transient downregulation of the catalytic activity of the nanoparticles.

In more general terms, we have developed straightforward methodology to gain temporal control over supramolecular processes, that is, all these processes rely on noncovalent interactions between molecules. In our particular case, ATP shifts the equilibrium between Au NP **1** and **A** away from the free energy minimum and continues to do so as long as its concentration remains above a certain threshold value. Although we have focused on transient signal generation, one can envision that the same strategy may also be applied to control the temporal stability of self-assembled systems, such as nanoarchitectures or materials, in which the degradable compound plays an essential role as a building block or as a stabilizing unit. As such, it may provide an important new tool for the design of synthetic systems with ‘life-like' properties, such as adaptation, self-healing and evolution.

## Methods

### General

Au NPs **1** were synthesized and characterized as described in the literature^24^ and stored at 4 °C in mQ water. The concentration of TACN-head groups was determined from kinetic titrations using either Zn(NO_3_)_2_ or Cu(NO_3_)_2_ as reported previously^25^. Zn(NO_3_)_2_ and Cu(NO_3_)_2_ were analytical grade products. The concentrations of metal ion stock solutions were determined by atomic absorption spectroscopy.

The synthesis and characterization of probes **A** (C343-GDD-OH)^31^ and **B** (Ac-WD-OH)^40^ have been reported previously. HPNPP was prepared as the sodium salt according to literature procedures^41^,^42^. The buffer, 4-(2-hydroxyethyl)-1-piperazineethanesulfonic acid (HEPES), ATP, ADP, AMP and potato apyrase were purchased from Sigma-Aldrich (product code A6237) and used without further purification. Na_3_PO_4_ (that is, P_i_) and CaCl_2_ were obtained from Carlo Erba Reagenti and BDH Prolabo, respectively, and used as received.

The enzyme potato apyrase was dissolved in 1.0 ml of mQ water and divided in 10 working aliquots of 100 μl, which were stored at −20 °C. To avoid harmful freeze–thaw cycles, all experiments were carried out using fresh solutions, which were prepared by diluting one of the abovementioned aliquots to the desired concentration and used within 1 day.

The enzyme concentration is expressed in U ml^−1^ and is based on the product information declared for the batch purchased (100 U). One unit is defined as the quantity of enzyme that will liberate 1.0 μmol of P_i_ from ATP or ADP per minute at pH 6.5 and 30 °C.

Stock solution concentrations of probes and competitors were determined both by weight and ultraviolet–visible spectroscopy using the following molar extinction coefficients: *ɛ*_259 (ATP, ADP, AMP)_=15,400 M^−1^ cm^−1^ and *ɛ*_450 (C343)_=45,000 M^−1^ cm^−1^ at pH 7.0)^43^,^44^.

Ultraviolet–visible measurements were performed on a Varian Cary 50 spectrophotometer, while fluorescence measurements were performed on a Varian Cary Eclipse fluorescence spectrophotometer. Both spectrophotometers were equipped with thermostatted cell holders. All experiments were performed starting from aqueous buffered solution ([HEPES]=10 mM, pH 7.0) containing Au NP **1** ([TACN·Zn^2+^]=10 or 20±1 μM). In a typical titration, consecutive amounts of stock solution of probes or competitors are added and the FI monitored as a function of time. Measurements were taken every minute. Optical parameters are reported in the figure captions.

### Complex formation between Au NP 1 and A or B

The SSC of **A** on Au NP **1** was determined from a fluorescence titration of **A** to Au NP **1** (10 μM). The FI at 493 nm was measured as a function of the amount of **A** added (see Supplementary Fig. 1a). The resulting curve was fitted using a model in which the Au NP **1** was represented by a single binding site with an imposed very high affinity (*K*_a_=1 × 10^8 ^M^−1^) for **A** to mimic binding under saturation conditions (see Supplementary Fig. 1b and Supplementary Data 1). A factor X was used to correlate the FI to the concentration of **A**. Fitting of the experimental data yielded a SSC of 3.7 (± 0.1) μM. The same value can also be obtained by extrapolation of the linear part to FI=0. The same procedure has been used to determine the SSC of **B** (see Supplementary Fig. 6) yielding a value of 0.6 (± 0.1) μM.

### Compatibility with potato apyrase and Ca^2+^

Potato apyrase requires metal ions for its hydrolytic activity (ATP→AMP+2P_i_), typically Ca^2+^ ions. To test the stability of the complex between Au NP **1** and **A** in the presence of Ca^2+^ and potato apyrase, the change in FI at 493 nm on the addition of increasing amounts of either CaCl_2_ or potato apyrase to a solution of Au NP **1** (10 μM) and probe **A** (3.7 μM) was monitored. Au NP **1**·**A** complex is hardly affected by the presence of both CaCl_2_ (up to 1.0 mM, see Supplementary Fig. 2a) and potato apyrase (at a fixed [CaCl_2_]=1.0 mM, see Supplementary Fig. 2b). Based on these results, it was decided to carry out all experiments at a constant concentration of CaCl_2_ (1.0 mM), which is sufficient for potato apyrase.

### Displacement studies

The relative affinities of probe **A** and a series of phosphate probes for Au NP **1** were determined from a series of competition experiments. Displacement studies were performed by measuring the FI at 493 nm on the addition of increasing amounts of either one of the competitors ATP, ADP, AMP, P_i_ or the combined mixture AMP+2P_i_ to a solution of Au NP **1** (10 μM) and probe **A** (3.7 μM) (see Supplementary Fig. 3). A theoretical model was developed to describe the competition experiments between probe **A** and the competitors ATP, ADP, AMP, P_i_ or the combined mixture AMP+2P_i_ (see Supplementary Fig. 4 and Supplementary Data 2). In the model, Au NP **1** is treated as having a single binding site for interaction with either one of the molecules. The total concentration of this binding site is equal to the SSC of **A** on Au NP **1** ([Au NP **1**]_tot_=3.7** **μM). The binding of probe **A** and the competitor to Au NP **1** is defined by the two thermodynamic equilibrium constants *K*_a_ and *K*_b_, respectively. A parameter X is used to correlate the concentration of unbound **A** to the FI (FI=*X* × **A**). As for the direct titrations, binding occurs under saturation conditions implying that the concentration of free Au NP **1** is very low. Consequently, the absolute values for *K*_A_ and *K*_B_ obtained from fitting the experimental data are not very informative. On the contrary, the ratio between *K*_A_ and *K*_B_ (*K*_rel_) is obtained very precisely as it depends on the ratio between free **A** and the amount of competitor added.

### Kinetic model

A theoretical model was developed to describe the kinetics of the system (see Supplementary Fig. 5 and Supplementary Data 3). In the model, Au NP **1** is treated as having a single binding site for interaction with **A**, ATP and P. The total concentration of this binding site is equal to the SSC of **A** on Au NP **1** ([Au NP **1**]_tot_=3.7** **μM). Starting point of the time course is an equilibrium system between **A** and Au NP **1** ([Au NP **1**·**A**]=3.47 μM, [**A**]_0_=[Au NP **1**]_0_=0.23 μM) to which ATP is added at *t*=0. This takes into account that a small amount of **A** is not bound when **A** is present at the SSC (see the binding curve in Supplementary Fig. 1). Three species interact with Au NP **1**: probe **A**, ATP and P, the latter representing the combined products of ATP hydrolysis (AMP+2P_i_). It is assumed that all associative processes are very fast (*k*_a_=10^7^) and that differences in thermodynamic stabilities arise from variations in the dissociation rate constants (*k*_d_). Thus, the different affinities of **A**, ATP and P for Au NP **1** are reflected by different dissociation constants (*k*_d,**A**_, *k*_d,**ATP**,_ *k*_d,**P**_, respectively). The value for *k*_d,**A**_ was arbitrarily set to a value of 2.5 to define binding of **A** to Au NP **1** under saturation conditions. This value is also sufficiently high to ensure a rapid release of probe **A** from Au NP **1** on the addition of ATP, as observed experimentally. The value of 2.5 for *k*_d,**A**_ also fixes the dissociation rate constants for ATP (*k*_d,**ATP**_) and P (*k*_d,**P**_), because the relative affinities of the species for Au NP **1** are known from the abovementioned displacement studies. Michaelis–Menten enzyme kinetics is implemented through a reversible interaction of ATP with the enzyme characterized by the association constant*, k*_a_, and the dissociation constant, *k*_d,ATP·enz_. Formation of the ATP–enzyme complex is followed by a conversion of ATP into products P (*k*_cat_), which then dissociate from the enzyme (defined by *k*_d,P·enz_). Development of the model showed that enzyme inhibition by product P is essential for a correct description of the kinetic profile. On the other hand, the introduction of ADP as reaction intermediate is not relevant from a kinetic point of view. The concentration of enzyme was set to an arbitrary value since its actual concentration is not known as the commercial solution is defined by enzyme activity, that is, the amount of ATP (nmol) that is consumed per min per mg. This implies that the values obtained for *k*_cat_ have no quantitative relevance. The low value of 0.01 μM for [*E*]_0_ (compared with Au NP **1**) ensures that the enzyme cannot accommodate large quantities of ATP. The FI is obtained by multiplying the concentration of free **A** with a factor X.

### Catalytic transphosphorylation of HPNPP by Au NP 1

The catalytic activity of Au NP **1** was evaluated by measuring the increase in absorbance at 400 nm (resulting from the formation of *p*-nitrophenolate) at different concentrations of HPNPP. the obtained kinetic data were elaborated with the method of initial rates. Initial rates (*ν*_0_) were then plotted against the concentration of HPNPP. The resulting enzyme-like saturation profile was fitted with the Michaelis–Menten model, from which the *k*_cat_ and *K*_m_ values are determined (See Supplementary Fig. 7).

## Additional information

**How to cite this article:** Pezzato, C. & Prins, L. J. Transient signal generation in a self-assembled nanosystem fuelled by ATP. *Nat. Commun.* 6:7790 doi: 10.1038/ncomms8790 (2015).

## Supplementary Material

Supplementary Information Supplementary Figures 1-8.

Supplementary Data 1 MicroMath Scientist Model File containing the model used for fitting of the binding isotherms of fluorophores to Au NP 1.

Supplementary Data 2 MicroMath Scientist Model File containing the model used for fitting of the displacement curves of fluorophores from Au NP 1 by a competitor

Supplementary Data 3 MicroMath Scientist Model File containing the model used for fitting transient signal generation.
