# Supplementary material for: Transient signal generation in a self-assembled nanosystem fueled by ATP
Source: Nat Commun. 2015 Jul 21;6:7790. doi: 10.1038/ncomms8790 (PMC4518249; doi:10.1038/ncomms8790)
Supplement: Supplementary Information — Supplementary Figures 1-8. [file ncomms8790-s1.pdf]

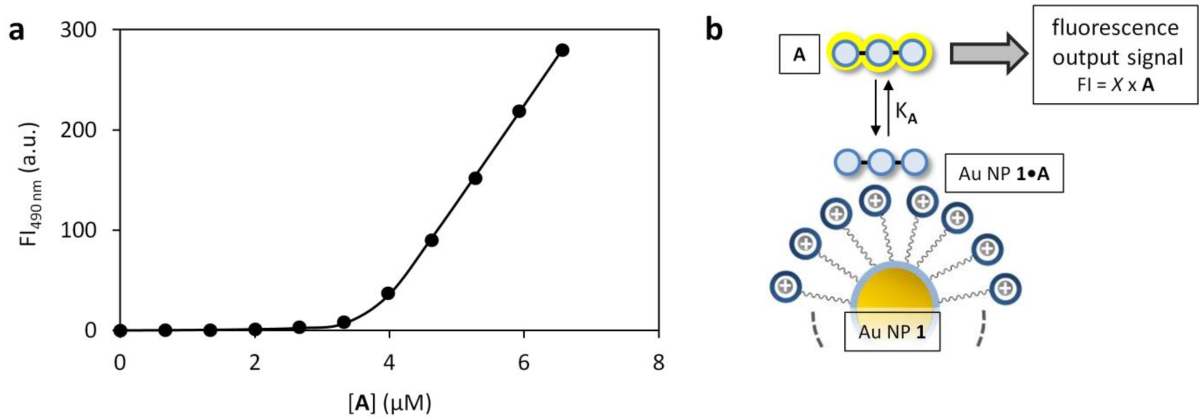

Supplementary Figure 1 | Complex formation between Au NP 1 and A. a) Fluorescence intensity at 493 nm as a function of the concentration of **A**. The solid line represents the best fit to the model depicted on the right. b) Thermodynamic model used to determine the surface saturation concentration of **A** on Au NP **1** (see Supplementary Data 1 for the corresponding MicroMath Scientist file). Experimental conditions: [TACN•Zn^{2+}] = 10 ± 1 µM, [HEPES] = 10 mM, pH = 7.0, T = 37 °C, λ_{ex} = 450 nm, λ_{em} = 493 nm (slits 2.5/5.0 nm).

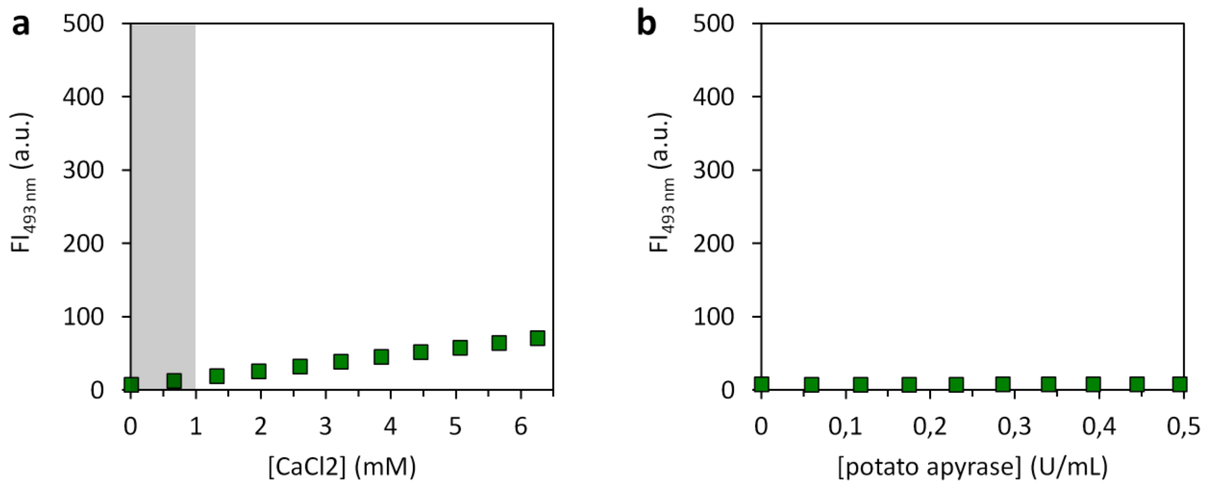

Supplementary Figure 2 | Compatibility of the Au NP 1•A complex with PA and Ca^{2+}. a) Fluorescent intensity at 493 nm as a function of the concentration of $CaCl_2$. Experimental conditions: [TACN•Zn^{2+}] = 10 ± 1 µM, [**A**] = 3.7 µM, [HEPES] = 10 mM, pH = 7.0, T = 37 °C. b) Fluorescent intensity at 493 nm as a function of the concentration of potato apyrase. Experimental conditions: [TACN•Zn^{2+}] = 10 ± 1 µM, [**A**] = 3.7 µM, [$CaCl_2$] = 1.0 mM, [HEPES] = 10 mM, pH = 7.0, T = 37 °C. λ_{ex} = 450 nm, λ_{em} = 493 nm (slits 2.5/5.0 nm).

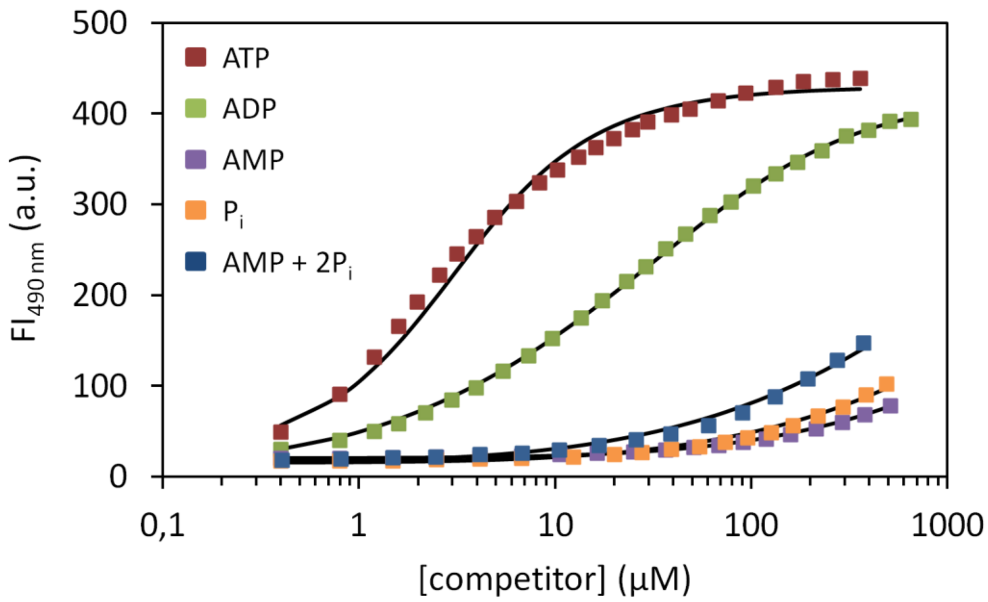

Supplementary Figure 3 | Displacement studies. Fluorescent intensities at 493 nm as a function of the concentration of added competitor. The solid lines represent the best fits to the thermodynamic model depicted in Supplementary Fig. 4. Experimental conditions: [TACN•Zn^{2+}] = 10 ± 1 µM, [**A**] = 3.7 µM, [$CaCl_2$] = 1.0 mM, [HEPES] = 10 mM, pH = 7.0, T = 37 °C, λ_{ex} = 450 nm, λ_{em} = 493 nm (slits 2.5/5.0 nm).

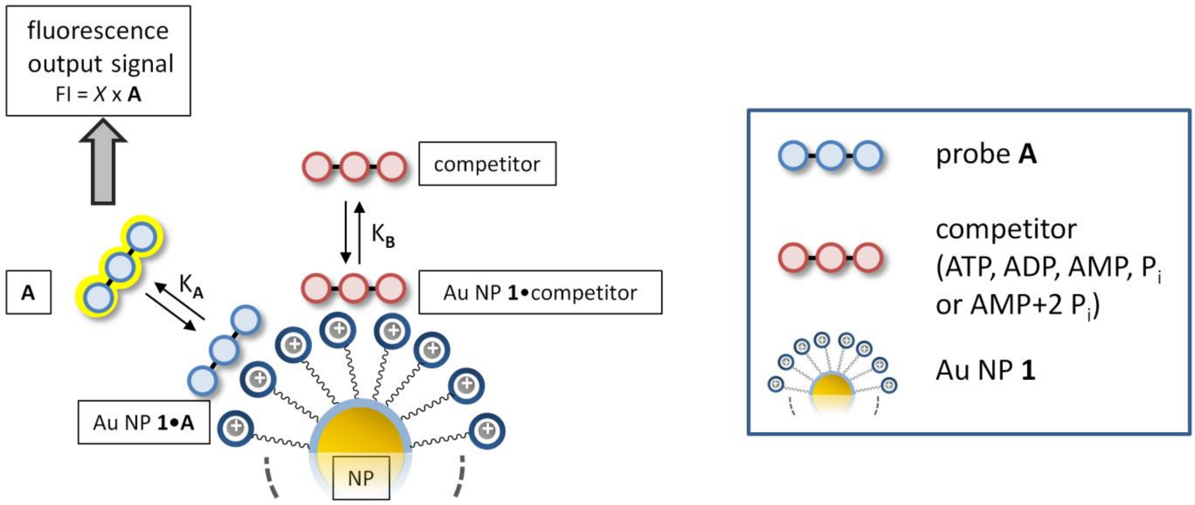

Supplementary Figure 4 | Schematic representation of the competition model. Thermodynamic model used to quantify the relative affinities of probe **A** and a competitor for Au NP **1** (see Supplementary Data 2 for the corresponding MicroMath Scientist file).

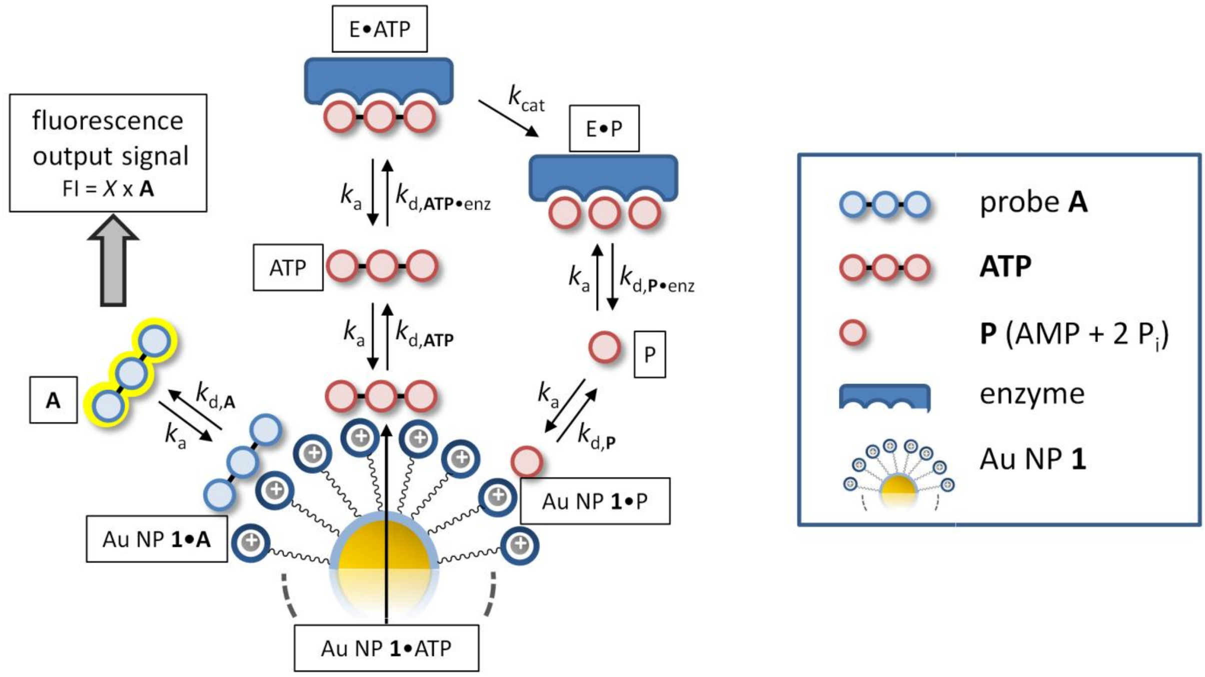

Supplementary Figure 5 | Schematic representation of the kinetic model. Kinetic model used to describe the fluorescence intensity as a function of time upon the addition of ATP (see Supplementary Data 3 for the corresponding MicroMath Scientist file).

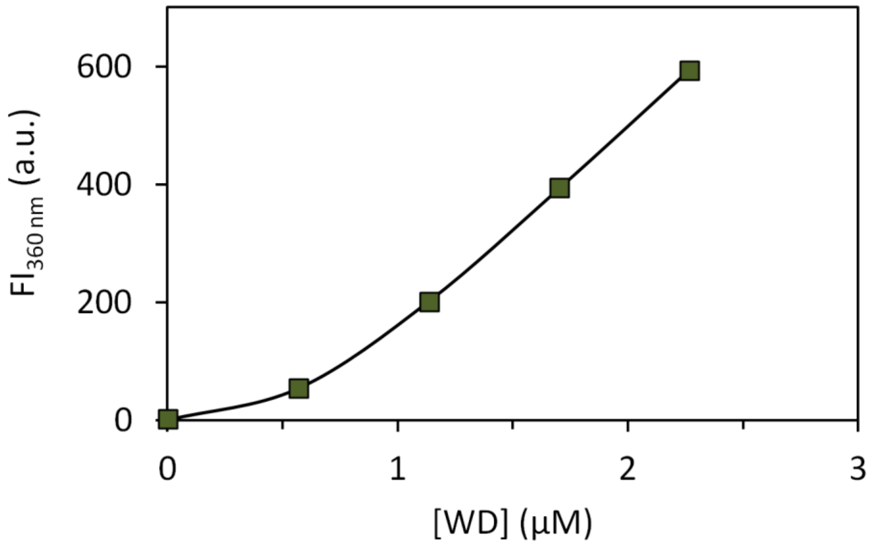

Supplementary Figure 6 | Complex formation between Au NP 1 and B. Fluorescence intensity at 360 nm as a function of the concentration of **B**. Experimental conditions: [TACN•Zn^{2+}] = 10 ± 1 µM, [HEPES] = 10 mM, pH = 7.0, T = 37 °C, λ_{ex} = 280 nm, λ_{em} = 360 nm (slits 10/10 nm).

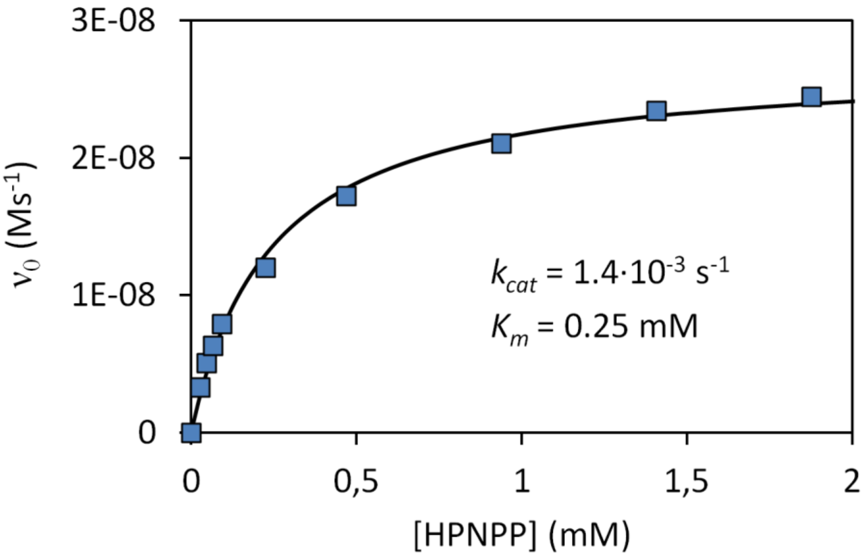

Supplementary Figure 7 | Catalytic transphosphorylation of HPNPP by Au NP 1. Initial rates for the transphosphorylation of HPNPP as a function of the concentration of HPNPP. The solid lines represent the best fits to the Michaelis-Menten model. Experimental conditions: [TACN•Zn^{2+}] = 20 ± 1 μM, HPNPP = 10 mM, [HEPES] = 10 mM, pH = 7.0, T = 40 °C.

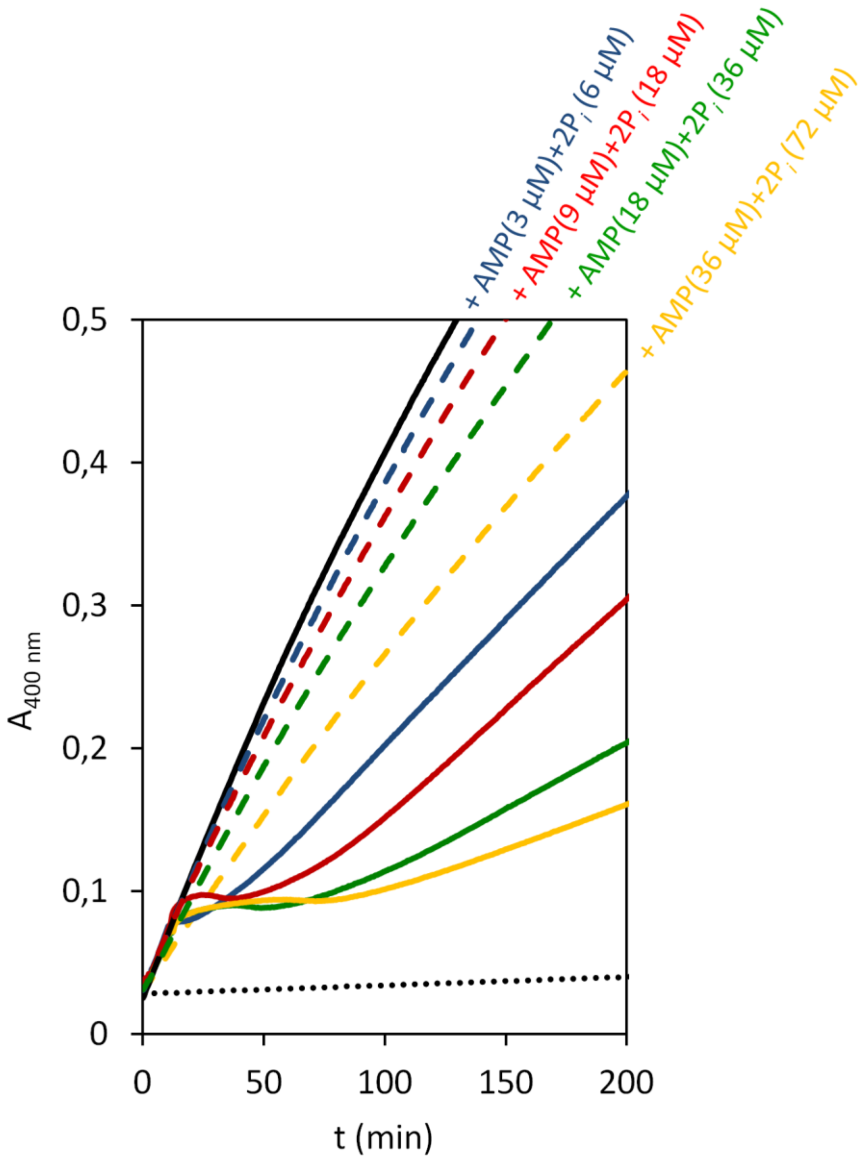

Supplementary Figure 8 | Transient down-regulation of catalytic activity. Absorbance at 400 nm (originating from the reaction product *p*-nitrophenolate) as a function of time in the absence of ATP (solid black line), in the presence of different concentrations of waste mixtures (colored dashed lines), in the presence of different concentration of ATP (solid colored lines) and enzyme and in the presence of ATP (3 µM) but without enzyme (dotted black line). Experimental conditions: [TACN•Zn^{2+}] = 10 ± 1 µM, [HPNPP] = 1 mM, [HEPES] = 10 mM, pH 7.0, [$CaCl_2$] = 1.0 mM, [potato apyrase] = 0.06 U/mL, 37 °C.
