# Supplementary material for: Transient signal generation in a self-assembled nanosystem fueled by ATP
Source: Nat Commun. 2015 Jul 21;6:7790. doi: 10.1038/ncomms8790 (PMC4518249; doi:10.1038/ncomms8790)
Supplement: Supplementary Data 1 — MicroMath Scientist Model File containing the model used for fitting of the binding isotherms of fluorophores to Au NP 1. [file ncomms8790-s2.docx]

// MicroMath Scientist Model File

//

// FI = fluorescence intensity; X = factor to correlate [**A**] to the fluorescence intensity (free)

// A = [**A**]; NP = [Au NP **1**]; NPA = [Au NP **1**•**A**]

//

//variables and parameters

IndVars: A0

DepVars: FI, A, NP, NPA

Params: X, K, NP0

// model

NPA=K*NP*A

A=A0-NPA

NP=NP0-NPA

FI=X*A

// constraints

0<A<A0

0<NP<NP0

0<NPA<NP0

***
