# Supplementary material for: Transient signal generation in a self-assembled nanosystem fueled by ATP
Source: Nat Commun. 2015 Jul 21;6:7790. doi: 10.1038/ncomms8790 (PMC4518249; doi:10.1038/ncomms8790)
Supplement: Supplementary Data 2 — MicroMath Scientist Model File containing the model used for fitting of the displacement curves of fluorophores from Au NP 1 by a competitor [file ncomms8790-s3.docx]

// MicroMath Scientist Model File

//

// FI = fluorescence intensity; X = factor to correlate [**A**] to the fluorescence intensity (free)

// A = [**A**]; B = [competitor]; NP = [Au NP **1**]; NPA = [Au NP **1**•**A**]; NPB = [Au NP **1**•competitor]

//

//variables and parameters

IndVars: B0

DepVars: FI, NPA, NPB, NP, A, B

Params: KA, KB, X, NP0, A0

// model

NPA=KA*NP*A

NPB=KB*NP*B

NP=NP0-NPA-NPB

A=A0-NPA

B=B0-NPB

FI=X*A

//boundaries

0<NPA<NP0

0<NPB<NP0

0<NP<NP0

0<A<A0

0<B<B0

***
