# Supplementary material for: Transient signal generation in a self-assembled nanosystem fueled by ATP
Source: Nat Commun. 2015 Jul 21;6:7790. doi: 10.1038/ncomms8790 (PMC4518249; doi:10.1038/ncomms8790)
Supplement: Supplementary Data 3 — MicroMath Scientist Model File containing the model used for fitting transient signal generation. [file ncomms8790-s4.docx]

// MicroMath Scientist Model File

//

// FI = fluorescence intensity; X = factor to correlate [**A**] to the fluorescence intensity (free)

// A = [**A**]; ATP = [ATP]; P = [P]

// NP = [Au NP **1**]; NPA = [Au NP **1**•**A**]; NPATP = [Au NP **1**•ATP]; NPP = [Au NP **1**•P]

// E = [E]; EATP = [E•ATP]; EP = [E•P]

// ka = *k*_a_ = 1x10^7^ (fixed); kda = *k*_d,_**_A_** = 2.5 (fixed); fatp = *k*_d,_**_A_**/*k*_d,ATP_ = 1.79 (fixed); fp = *k*_d,_**_A_**/*k*_d,P_ = 0.00163 (fixed)

// kd = *k*_d,ATP•E_  (free); kdep = *k*_d,P•E_ (free); kcat = *k*_cat_ (free)

//

//variables and parameters

IndVars: T

DepVars: FI, NPA, NPATP, NPP, NP, A, ATP, P, EATP, E, EP, NPTOT, ETOT

Params: ka, kda, fatp, fp, kd, kdep, kcat, X, NPA0, A0, NP0, ATP0, E0

//model

kdatp=kda/fatp

kdp=kda/fp

NPA'=ka*NP*A-kda*NPA

NPATP'=ka*NP*ATP-kdatp*NPATP

NPP'=ka*NP*P-kdp*NPP

NP'=-ka*A*NP+kda*NPA-ka*ATP*NP+kdatp*NPATP-ka*NP*P+kdp*NPP

A'=-ka*A*NP+kda*NPA

ATP'=-ka*NP*ATP+kdatp*NPATP-ka*E*ATP+kd*EATP

EATP'=ka*E*ATP-kd*EATP-kcat*EATP

EP'=ka*E*P-kdep*EP

E'=-ka*E*ATP+kd*EATP+kcat*EATP-ka*E*P+kdep*EP

P'=-ka*P*NP+kdp*NPP+kcat*EATP-ka*E*P+kdep*EP

FI=X*A

//initial conditions

T=0

NPA=NPA0

NPATP=0

A=A0

NP=NP0

ATP=ATP0

P=0

NPP=0

E=E0

EP=0

EATP=0

***
